# The interaction between children and the environment: an agenda for health

**DOI:** 10.1590/0102-311XEN232025

**Published:** 2026-03-16

**Authors:** Tatiana Vasconcelos dos Santos, Martha Cristina Nunes Moreira

**Affiliations:** 1 Universidade Federal do Rio de Janeiro, Rio de Janeiro, Brasil.; 2 Instituto Nacional de Saúde da Mulher, da Criança e do Adolescente Fernandes Figueira, Fundação Oswaldo Cruz, Rio de Janeiro, Brasil.

On the eve of COP30, we evoke an agenda on child health in light of the Social Determinants of Health ^1^, integrating a countercolonial critique based on Biointeractions ^2^ and interdependence as a nature-related human condition ^3^.

The expropriation and the induction of inequalities caused by the capitalist system yield a nature-humanity binomial and an understanding of the environment as a source of natural resources to be exploited based on their utility. This logic has repercussions on how health is addressed and produced, making increasingly evident the impossibility of this social system and the mode of civilization it establishes being capable of building reciprocal relationships with nature and healthy ways of life.

Children are extensively affected by the way capital alters our relationship with nature, constituting a population impacted by the climate crisis and by crimes related to environmental exploitation, by companies exploiting territories and forcing displacement, and by family disaffiliation. Children are expropriated from the right to childhood with protection, education, health, and leisure.

The 2022 United Nations Children Fund (UNICEF) report ^4^ notes that, in Brazil, 40 million girls and boys are exposed to more than one climate or environmental risk (60% of the total) and climate change compromises the guarantee of fundamental rights. It is acknowledged that rights violations occur and that public policies do not provide the necessary recognition to different groups of vulnerable children (*quilombola*, with disabilities, Black, Indigenous, migrant/refugee, from traditional communities).

The repercussions of climate disasters and emergencies are long-lasting, and they refer to a sensitive place of family, community, and socio-environmental affiliations that are so relevant to the health of children and adolescents. Jaime Breilh ^1^ assumes the debate on affiliation and disaffiliation as strategic within the Social Determinants of Health. Less as a variable to be described, the social situations of gender, generation, territory, race, ethnicity, and ability refer to the distribution of power, expropriation, and exploitation. A destroyed, expropriated, exploited nature, forgotten by State policies and care, transforms into insecurity.

Also according to UNICEF ^4^, children and adolescents have their physical health disproportionately affected by changes in their living environments, with their development and well-being directly impacted by changes in temperature, air and water quality, and available nutrition means.

Aware of the climate crisis, children can perceive nature as a threat, which translates into a fear of rain, landslides, floods, and interactions with the environment. In the face of this scenario, we do not want to lose the opportunity to evoke an agenda for child health in light of Biointeractions ^2^ to be developed through a relationship of reciprocity, with nature as an intercessor, relational and interdependent.

In the late 2024, the Brazilian Society of Pediatrics (SBP, acronym in Portuguese) published an update document on the benefits of nature in child development ^5^. Based on a vast scientific literature, the material proposes strategies for interaction between children and nature and provides insights into how this can be a means for health promotion.

Discussions about the benefits of nature for children’s health are commonly found among health care providers, researchers, in the scientific literature, and in official documentation on the issue, as noted above in the SBP document. Despite acknowledging the importance of the biomedical perspective that provides us with evidence of these benefits for children's physical and emotional health, we propose considering the issue less from a therapeutic utility perspective and more from a relational perspective.

With Maria Puig de la Bellacasa and her feminist ethics of care, we propose considering the child-nature interrelation as a “matter of care” ^3^. Inspired by the concept of “matters of concern” of Latour ^6^, the author proposes “matters of care”, mobilizing marginalized and neglected elements. Turning something into a matter of care means relating to these elements, being affected by them, and evoking their potential to affect others.

Consistently with Puig de la Bellacasa, we propound that adequately addressing the issue of child-nature disconnection requires the production of knowledge that touches upon the subtleties related to the issue, shedding light on invisibilized aspects such as the elements irreducible to the individual and a space for interventions.

Our perspective is that the discourse of therapeutic utility arises from and feeds back into an implicit hierarchy between human and non-human life, a hegemonic and colonial perspective that nature is at our service.

As well argued by Ferdinand ^7^, colonial relations are not just translated into relations between human groups: they include relations with non-humans, landscapes, and spaces as a form of occupation. Our dwelling excludes the presence of natural landscapes, non-human beings, and relates to the perception of which bodies can access nature, configuring intersections with the categories of race, class, and ability.

In this context, thinking about the child-nature relationship as a matter of care invites us to consider the subtleties of a dialogue between disconnection and uncare practices, which include human and non-human subjects and inform our way of being and existing in the world. It is an attempt to overcome that which Ferdinand calls a “*detached sympathy*” when talking about environmental issues ^7^.

In this context, there arises the question: How much of the domination practices shown in the hierarchical relationships we weave with nature shape our behavior in other spheres, with reverberations in biomedical discourses, in child health policies, and in the way we think about the spaces in which children move?

The proposal is to examine carefully and consider with attention, interrelating historically divided aspects, building bridges in an attempt to correct fractures.

Thinking about expansions for the collective, addressing the issue of children's disconnection from nature, considering its fractures and urgencies, is a means of “*staying with the trouble*”, as note by Donna Haraway ^8^. Staying with the trouble leads us to another matrix of sensitivity production that unfolds into more attentive and careful observation of the world, research practices, and knowledge production. By proposing the discussion based on this conception, we believe that “something” can be found and that, from that, we may obtain insights into how to create more creative and potent health policies.

Seeking to be affected by the set of things that mobilize the problem of children’s disconnection from nature and its dialogue with health, we are interested in including historically excluded and neglected perspectives and subjects into the debate. We will bring into the conversation Indigenous and *quilombola* worldviews, as well as “ability” as a social marker of difference that ratifies a hegemonic discourse about who can or cannot be in nature.

We propose Biointeraction to think about the relations between children and nature. Antonio Bispo dos Santos, a *quilombola* thinker, proposes this category as a civilizational alternative in which the interaction between humans and nature is a matter of reciprocity, with sharing as an inherent characteristic ^2^. Interactions are governed by exchanges, not utility.

Children’s interactions with the textures, smells, sounds, and colors of nature provide possibilities for sensory connection that connect them to their non-human relatives, consistently with that which Krenak calls “*friction with life*” ^9^. Sensory experience as an exercise in bridging the distance with nature. Mediated by what is mineral and what is vegetal, children connect with life by understanding these elements as belonging to the vital essence within us.

When we think about the category of “ability”, we shed light on children with disabilities. For them, the possibility of friction with life through interaction with nature provides the possibility of a mutually constitutive contact, despite possible physical, sensory, or intellectual hindrances. Thus, there arises a possibility of breaking a hegemonic perspective in which abilities such as walking, hearing, and seeing are necessary to living legitimate experiences in nature. Thinking about the experiences of contact with nature of children with disabilities based on Biointeractions provides new pathways for our understanding of the non-human nature, blurring the very line between the human and the non-human.

All of this is already sufficient in itself. Adopting a therapeutic utility logic is not required to justify this relationship. Biointeraction provides the possibility of building stories of enchantment, mutual care, and the empowerment of other ways of existing and being in spaces in interaction with nature.

On the agenda of the disconnection between children and nature, the lack of access to green areas is a recurring issue in official documents. The lack of opportunity for children to connect with nature is considered a violation of rights guaranteed by the *Brazilian Federal Constitution* and by international treaties and conventions ^10^.

As a backdrop is the destructive way we inhabit spaces. Thinking of infrastructure as a matter of care leads us to consider: How are we configuring the spaces where children move? How has the marker of ability and its intersections with other social markers of difference determined which bodies can be in and with nature?

The document of the SBP ^5^ strives to indicate some important paths and actors in the discussion of access to nature for children with disabilities. However, in the dimension of access, it is worth considering the previous arguments of theorists in the field of disability studies ^11^ ^,^ ^12^. Focusing on accommodation with removal of environmental barriers to think about access is based on the assumption that disability is stable and knowable. As if we could say what people with disabilities will need, under the assumption that their needs are sufficiently stable, sufficiently predictable, without recognizing other social markers of difference that interrelate with the category of ability.

Data on how the climate crisis affects people with disabilities indicate the need to consider the intersection of vulnerabilities ^13^. Aspects such as social inequality and structural poverty interrelate with the lack of infrastructure for access to natural resources. In addition to being more vulnerable to the impacts of the climate crisis, this population has less access to green areas. These aspects should be on the agenda for discussions about child health and nature, demonstrating a commitment to an active process of intervening in the count of what and who are ratified as of interest.

Transforming things into matters of care is a way of relating to them, producing care and knowledge by finding means to resignify an objectified world ^3^. Adopting the category of Biointeraction to think about how we relate to nature and produce knowledge about child health can be a way to consider neglected aspects, taking from the world poetry to invent new worlds.

Conceiving care in this context is both an ethical-political practice and commitment that affects how we produce knowledge about things.

The invitation is for scientific production, official documents, professional discourses, and public policies on child health and nature to align with discussions on the social determinants of health, adopting an ethical-political commitment toward creating bonds with the invisibilized subjects, concerns, and aspects of the issue. This process has the disruptive potential to lead us to think about children's relationship with nature less from the perspective of therapeutic utility and more from the perspective of reciprocity, helping us to complexify the issue and to be attentive to which narratives gain more visibility.
