# Supplementary material for: The interaction between children and the environment: an agenda for health
Source: Cad Saude Publica. 2026 Mar 16;42:e00232025. doi: 10.1590/0102-311XEN232025 (PMC12995291; doi:10.1590/0102-311XEN232025)
Supplement: Translation

# Translation: A interação criança e ambiente: uma agenda para a saúde

**DOI:** 10.1590/0102-311XPT232025

**Authors:** Tatiana Vasconcelos dos Santos, Martha Cristina Nunes Moreira

**Affiliations:** 1 Universidade Federal do Rio de Janeiro, Rio de Janeiro, Brasil.; 2 Instituto Nacional de Saúde da Mulher, da Criança e do Adolescente Fernandes Figueira, Fundação Oswaldo Cruz, Rio de Janeiro, Brasil.

Às vésperas da COP30, provocamos uma agenda sobre a saúde da criança à luz da Determinação Social da Saúde ^1^, integrando uma crítica contracolonial baseada nas Biointerações ^2^ e na interdependência como condição humana relacionada à natureza ^3^.

A expropriação e a indução de desigualdades geradas pelo sistema capitalista produzem um binômio natureza e humanidade e uma compreensão do ambiente como fonte de recursos naturais a serem explorados a partir da sua utilidade. Essa lógica apresenta reverberações sobre como se fala e se produz saúde, tornando-se cada vez mais patente a impossibilidade desse sistema social e o modo de civilização que ele instaura, serem capazes de construir relações de reciprocidade com a natureza e modos de vida saudáveis.

As crianças são extensamente afetadas pela forma com que o capital altera nossa relação com a natureza, conformando uma população atingida pela crise climática e por crimes relacionados a exploração ambiental, exploração por empresas de territórios que forçam deslocamentos e desfiliações em famílias. Crianças expropriadas do direito à infância com proteção, educação, saúde e lazer.

O relatório do Fundo das Nações Unidas para a Infância (UNICEF) de 2022 ^4^ destaca que, no Brasil, 40 milhões de meninas e meninos estão expostos a mais de um risco climático ou ambiental (60% do total) e as mudanças climáticas comprometem a garantia de direitos fundamentais. Há um reconhecimento de que ocorrem violações de direitos e de que as políticas públicas não oferecem o reconhecimento necessário aos diferentes grupos de crianças vulnerabilizadas (quilombolas, com deficiência, negras, indígenas, migrantes/refugiados, de comunidades tradicionais).

As repercussões dos desastres e emergências climáticas são duradouras, e remetem a um lugar sensível das filiações familiares, comunitárias e socioambientais tão relevantes para a saúde de crianças e adolescentes. Jaime Breilh ^1^ assume o debate sobre filiação e desfiliação como estratégico no interior da Determinação Social da Saúde. Menos como uma variável a ser descrita, as localizações sociais de gênero, geração, território, raça, etnia, capacidade remetem à distribuição de poder, expropriação e exploração. Uma natureza destruída, expropriada, explorada, esquecida pelas políticas e pelo cuidado do Estado se transmuta em insegurança.

Ainda segundo o UNICEF ^4^, crianças e os adolescentes têm suas saúdes físicas afetadas de forma desproporcional por mudanças nos ambientes em que vivem, tendo seu desenvolvimento e bem-estar afetados diretamente por mudanças na temperatura, na qualidade do ar e da água e nos meios disponíveis para a nutrição.

Conscientes sobre a crise climática, as crianças podem ler a natureza como ameaça que se traduz no medo da chuva, do deslizamento, das enchentes e das interações com o meio ambiente. Frente a este cenário, não queremos perder a possibilidade de provocar uma agenda para a saúde da criança à luz das Biointerações ^2^ a se desenvolver a partir de uma relação de reciprocidade, tendo a natureza como intercessora, relacional e interdependente.

No segundo semestre de 2024, a Sociedade Brasileira de Pediatria (SBP) publicou um documento de atualização sobre os benefícios da natureza no desenvolvimento de crianças ^5^. Apoiando-se em uma vasta literatura científica, o material propõe estratégias para que a interação criança e natureza aconteça e nos fornece pistas de como esse pode ser um caminho para a promoção da saúde.

Os discursos sobre os benefícios da natureza para a saúde da criança circulam comumente entre os profissionais de saúde, pesquisadores, na literatura científica e nos documentos oficiais sobre o tema, como assinalado acima citando o documento da SBP. Embora considerando a importância da perspectiva biomédica que nos fornece evidências desses benefícios para a saúde física e emocional das crianças, propomos pensar sobre o tema menos pela utilidade terapêutica e mais pela perspectiva relacional.

Com Maria Puig de la Bellacasa e sua ética feminista do cuidado, propomos pensar a interlocução criança e natureza como “questão de cuidado” (*matters of care*) ^3^. Inspirada pelo conceito de “questões de interesse” (*matters of concern*) de Latour ^6^, a autora propõe as “questões de cuidado” mobilizando elementos marginalizados e negligenciados. Tornar algo uma questão de cuidado significa relacionar-se com esses elementos, afetar-se por eles e evocar seu potencial de afetar os outros.

Seguindo Puig de la Bellacasa, nossa aposta é que, para nos envolvermos adequadamente com o problema da desconexão criança e natureza, precisamos produzir conhecimento que toque nas sutilezas relacionadas ao tema, colocando luz sobre aspectos invisibilizados como os elementos irredutíveis ao indivíduo e um espaço para intervenções.

Nossa perspectiva é que o discurso da utilidade terapêutica nasce de e retroalimenta uma hierarquia implícita entre a vida humana e não humana, uma perspectiva hegemônica e colonial de que a natureza está a nosso serviço.

Como bem coloca Ferdinand ^7^, as relações coloniais não se traduzem apenas nas relações entre grupos humanos: compreendem relações com não humanos, paisagens e espaços como uma forma de ocupação. O nosso habitar exclui a presença de paisagens naturais, seres não humanos e se relaciona com a percepção de quais corpos podem ter acesso a natureza, configurando intersecções com as categorias de raça, classe e capacidade.

Nesse contexto, pensar a relação criança e natureza como questão de cuidado nos convida a considerar as sutilezas de um diálogo entre as práticas de desconexão e descuidado, que incluem sujeitos humanos e não humanos e conformam nossa forma de ser e estar no mundo. Uma tentativa de superar o que Ferdinand chama de uma “*simpatia sem vínculo*” quando se fala sobre questões ambientais ^7^.

Nesse contexto, cabe o questionamento: Quanto das práticas de dominação mostradas nas relações hierarquizadas que tecemos com a natureza moldam nosso comportamento em outras esferas, com reverberações nos discursos biomédicos, nas políticas de saúde para a criança e na forma como pensamos os espaços em que a criança circula?

A proposta é olhar com cuidado e prestar atenção, fazendo aproximações de aspectos historicamente cindidos, construindo pontes na tentativa de corrigir fraturas.

Pensando em expansões para o coletivo, abordar o tema da desconexão da criança com a natureza considerando suas fraturas e urgências é uma forma de “*ficar com o problema*”, como aponta Donna Haraway ^8^. Ficar com o problema nos leva para uma outra matriz de produção de sensibilidade que se desdobra em observação do mundo, práticas de pesquisa, e produção de conhecimento mais atentas e cuidadosas. Ao propor a discussão a partir dessa concepção acreditamos que “algo” pode ser encontrado e que, a partir disso, talvez tenhamos pistas de como criar políticas de saúde mais criativas e potentes.

Buscando nos afetar pelo conjunto de coisas que mobilizam o problema da desconexão da criança com a natureza e seu diálogo com a saúde, nos interessa incluir no debate perspectivas e sujeitos historicamente excluídos e negligenciados. Traremos para a conversa as cosmovisões indígenas e quilombolas e a “capacidade” como marcador social da diferença que ratifica um discurso hegemônico de quem pode ou não estar na natureza.

Propomos a Biointeração para pensarmos as relações criança e natureza. Antonio Bispo dos Santos, pensador quilombola, propõe essa categoria como uma alternativa civilizatória na qual a interação entre humanos e natureza é da ordem da reciprocidade, guardando o compartilhamento como característica inerente ^2^. O que regem as interações são as trocas e não a utilidade.

As interações da criança com as texturas, os cheiros, os sons e as cores da natureza abrem possibilidades de conexão sensorial que a liga aos seus parentes não humanos, se aproximando do que Krenak chama de “*fricção com a vida*” ^9^. A experiência sensorial como exercício de transpor a distância com a natureza. Mediados pelo que é mineral e pelo que é vegetal, a criança se conecta com a vida entendendo esses elementos como pertencentes à essência vital que há em nós.

Quando pensamos na categoria “capacidade” lançamos luz sobre as crianças com deficiência. Para elas, a possibilidade de fricção com a vida através da interação com a natureza abre a possibilidade de um contato mutuamente constituinte, a despeito de possíveis impedimentos físicos, sensoriais ou intelectuais. Abre-se, assim, uma possibilidade de quebra de uma perspectiva hegemônica em que habilidades como andar, ouvir, ver são necessárias para viver experiências legitimadas na natureza. Pensar as experiências de contato de crianças com deficiência com a natureza tendo as Biointerações como pano de fundo abre novos caminhos para a nossa compreensão em relação à natureza não humana, borrando a própria linha entre o humano e o não humano.

Tudo isso já se basta em si. Não há necessidade de acionar uma lógica de utilidade terapêutica para justificar essa relação. A Biointeração traz a possibilidade de construção de estórias de encantamento, cuidado mútuo e potencialização de outros modos de existir e de estar nos espaços em interação com a natureza.

Na pauta da desconexão criança e natureza, a falta de acesso às áreas verdes é uma discussão recorrente nos documentos oficiais. A carência de oportunidade de contato da criança com a natureza é considerada como uma violação de direitos garantidos pela *Constituição Federal* e por tratados e convenções internacionais ^10^.

Como pano de fundo está a forma destrutiva com que habitamos os espaços. Pensar na infraestrutura como questão de cuidado nos convoca a pensar: Como estamos configurando os espaços por onde as crianças circulam? Como o marcador da capacidade e as intersecções com outros marcadores sociais da diferença tem determinado quais corpos podem estar na e com a natureza?

O documento da SBP ^5^ esforça-se para apontar alguns caminhos e atores importantes na discussão do acesso de crianças com deficiência à natureza. Porém, na dimensão do acesso, cabe considerar a provocação já feita por teóricas do campo dos estudos da deficiência ^11^ ^,^ ^12^. Focar na acomodação com retirada de barreiras ambientais para pensar o acesso baseia-se na suposição de que a deficiência é estável e cognoscível. Como se pudéssemos dizer o que as pessoas com deficiência irão precisar, em um entendimento que as necessidades são suficientemente estáveis, suficientemente previsíveis, não reconhecendo outros marcadores sociais da diferença que dialogam com a categoria de capacidade.

Dados relativos a como a crise climática afeta pessoas com deficiência apontam para a necessidade de se pensar na intersecção de vulnerabilidades ^13^. Aspectos como desigualdade social e pobreza estrutural dialogam com a falta de uma infraestrutura acessível a bens naturais. Além de serem mais vulneráveis aos impactos da crise climática, essa população tem menos possibilidade de acesso às áreas verdes. Esses aspectos devem estar na pauta da discussão sobre saúde da criança e natureza, evidenciando o comprometimento com um processo ativo de intervir na contagem do que e quem são ratificados como de interesse.

Transformar coisas em questões de cuidado é uma maneira de se relacionar com elas, produzir cuidado e conhecimento encontrando maneiras de reafetar um mundo objetificado ^3^. Trazer a categoria de Biointeração para pensar a forma como nos relacionamos com a natureza e produzimos conhecimento sobre saúde da criança pode ser uma forma de prestar atenção em aspectos negligenciados, tirando do mundo poesias para inventar novos mundos.

Conceber o cuidado nesse contexto é tanto um fazer quanto um compromisso ético-político que afeta a maneira como produzimos conhecimento sobre as coisas.

O convite é para que a produção científica, os documentos oficiais, os discursos dos profissionais e as políticas públicas sobre o tema saúde da criança e natureza se alinhem às discussões sobre determinação social da saúde, assumindo um compromisso ético-político de criação de vínculo com os sujeitos, as preocupações e aspectos invisibilizados sobre o tema. Esse processo tem o potencial disruptivo para pensarmos a relação da criança com a natureza menos pela perspectiva da utilidade terapêutica e mais pela perspectiva da reciprocidade, nos ajudando a complexificar o problema e a estarmos atentos a quais narrativas ganham mais visibilidade.
